# Politics

**Published:** 1884-10

**Authors:** J. B. Chandler


### POLITICS.

J. B. CHANDLER.

Our language seems to lack words, and we find in many instances the same nouns used to express things which bear but a very indirect relation to one another. This may be accounted for in the fact of the lexicographer, in his anxiety to keep up with the popular development of language, accepting and engrafting upon the later editions of his Dictionary, definitions which, beginning in slang, have gradually been adopted in general conversation. Thus we find Webster gives two definitions for the word politics.

1. "The science of government; that part of ethics which has to do with the regulation and government of a Nation or State, the preservation of its safety, peace, and prosperity; the defense of its existence and rights against foreign control or conquest, the augmentation of its strength and resources, and the protection of its citizens in their rights, with the preservation and improvement of their morals.',

2. "The management of a political party; the advancement of candidates to office; in a bad sense, artful or dishonest management to secure the success of political measures or party schemes; political trickery."

The great principle upon which a republican form of government is founded, the ability of the people to govern themselves, is a beautiful theory and in time may prove itself the best in practice for the whole world; but whilst ignorance and vice are powerful elements in so many communities trouble will arise, and until education shall have blotted out the one and limited if not obliterated the other, there will be enough citizens who are unfit to govern themselves to create disturbances and sometimes overbalance those who are truly within the postulate; and thus seem to prove that the general law is not correct but should be written "People of intelligence and moral honesty can govern themselves."

Of course the standard of intelligence and moral honesty which is required for the selection of good rulers is not beyond the reach of any outside of the pales of idiocy or insanity, but the day for realizing the full and perfect success of a republic based upon an enlarged elective franchise including the entire adult male population seems far distant.

*Vox populi* is as yet very, very far from being *Vox Dei.*

Let us consider the first and original definition of the word politics—embracing as it does all that a patriot holds sacred in considering his country, all in that relation, which ought to be of interest to the immigrant who has left his fatherland and engrafted the fortunes of himself and his progeny in a new country.

Every citizen is certainly deeply interested in the formation or perpetuation of a good government and in the preservation of the safety, peace and prosperity of the Nation, with the defense of its existence and rights against foreign control, &c.—these are the ends for which the true politician should labor, and these are questions which are of vital importance to all, from the new born babe to the Octogenerian. It seems but right that in matters of such general and personal interest all should be entitled to a say, either direct or representative.

In this view may be found the argument for the utmost extension of the elective franchise; but the dangers which assail the republic from such a privilege granted indiscriminately to the uneducated, irresponsible and vicious are to be considered.

In the midst now of a presidential campaign, with its excitements and anxieties, agitating the whole country to its utmost limits, (an evil repeated every four years,) embracing questions of policy involving the welfare of over fifty millions of human beings, it is sad to have to confess that Webster's second and later definition of the word politics maintains the ascendancy.

What a sad contemplation is suggested by torch-light processions, bands of music, fireworks, copper badges and all the accepted *political* paraphanalia deemed necessary for inducing the, so estimated, able and discriminating patriotic voter to elect a candidate who has ability to take the helm of State and carry out the great objects embodied in Webster's first definition of "Politics." What consideration of these great questions does the voter give who, brought up in poverty and ignorance under the grind of a strong government with all his capabilities of patriotism and religion absorbed in what he considers duties in another continent, who makes his cross to his naturalization papers and comes to the polls to vote? If he *does* give consideration, what attributes has he which should entitle his ballot to the same power in ruling the destinies of this country as that of one whose life has been spent in the study of political economy to be applied in this form of government and whose surroundings through life have been numbered with the spirit and genius of republican institutions? Again, the slave, who, brought up in carefully protected ignorance, is freed, and in a day given the privileges and power of a freeman, without any preparation whatever; expected to follow the lash of party discipline as he bended in other days to the lash of the overseer.

Is it to be wondered at, that in either of these cases the elements of religious bias, old country love, or race affiliations, should lead to combinations antagonistic to the spirit of our institutions and calculated if unrestrained to lead to their overthrow?

These elements although not to be despised, are less to be feared than the apathy of the better classes which abandons the political machinery to unscrupulous politicians (*vide*—Webster's second definition) whose pecuniary interests go hand in hand with party affiliations and whose patriotism is comprehended within the probable stealings to be gained whilst their man is in power.

Were it within the scope of a journal like the BISTOURY to particularize the apparent departure of both of the great parties now contending for supremacy in this country, from what would seem to be the proper method of eliciting and expressing the well digested opinion of the people upon the questions involved in the present contest, much could be held up to ridicule on both sides, and attention drawn to the methods and schemes used to gain power and votes by apparent leaning toward, or promises made in relation to, prejudices or conspiracies of certain classes, which, if carried out, would have little tendency towards "the preservation of the safety, peace and prosperity of the nation," but we forbear; and are thus saved possibly from having the reader accuse us of Eutopean schemes.

Perhaps necessity forces the use of such tools and perhaps in this instance the end may justify the means. Nevertheless the fact remains that unless some qualification is attached to the elective franchise and some restraint put upon the immigration of the worst elements from European and Asiatic countries and questions entirely foreign to the interest of our beloved country are kept out of political contests, the time will come when the pure element will cease to be powerful enough to deceive the people into governing themselves properly.

It is a pity that so beautiful a theory as we started with at the head of this article should require the use in practice, of such childish tools in the great work of self government, but if, as it seems, they are useful let us give freely towards, and loudly applaud, the drum, the gun, the rocket and the various boyish organizations that lead to the election of suitable officers.

There are optimists and there are pessimists amongst us, the former prophecying, grand and undying future for the great Republic, the latter through their smoky glasses seeing the writing on the wall. The capabilities of the country are with the prophecies of the former, the dangers are with ourselves.

